# Persistence in ESG and conventional stock market indices

**DOI:** 10.1007/s12197-022-09580-0

**Published:** 2022-05-07

**Authors:** Guglielmo Maria Caporale, Luis Gil-Alana, Alex Plastun, Inna Makarenko

**Affiliations:** 1 Department of Economics and Finance, Brunel University London, Uxbridge, UB8 3PH UK; 2 University of Navarra, Pamplona, Spain; 3 Sumy State University, Sumy, Ukraine

**Keywords:** Stock market, ESG, Persistence, Long Memory, R/S Analysis, Fractional Integration, C22, G12

## Abstract

This paper uses R/S (Rescaled Range) analysis and fractional integration techniques to examine the persistence of two sets of 12 ESG (Environmental, Social and Governance) and conventional stock price indices from the MSCI ((Morgan Stanley Capital International) database over the period 2007–2020 for a large number of both developed and emerging markets. Both sets of results imply that there are no significant differences between the two types of indices in terms of the degree of persistence and its dynamic behaviour. However, higher persistence is found for the emerging markets examined (especially the BRICS, i.e. Brazil, Russia, India, China and South Africa), which suggests that they are less efficient and thus offer more opportunities for profitable trading strategies. Possible explanations for these findings include different type of companies’ ‘camouflage’ and ‘washing’ (green, blue, pink, social, and Sustainable Development Goals—SDG) in the presence of rather lax regulations for ESG reporting.

## Introduction

In recent years ESG (Environmental, Social and Governance) analysis has become an important part of the investment process given the increasing attention being paid to the sustainability and societal impact of investing in a company or business. In contrast to traditional stock indices, ESG ones are based on social responsibility criteria to screen and select their components. According to the MSCI (Morgan Stanley Capital International) 2021 Global Institutional Investor survey (a survey of 200 asset owner institutions with assets totalling approximately $18 trillion) over three-quarters (77%) of investors increased ESG investments ‘significantly’ or ‘moderately’ in 2020, with this figure rising to 90% for the largest institutions (over $200 billion of assets). The corresponding percentages were 79% for the Asia–Pacific region, 78% for the US and 68% for the EMEA (Europe, Middle East and Africa) group of countries.^1^ Also, over $19 billion flowed into ESG ETFs (Exchange Traded Funds) in 2020 (up from $8 billion in 2019), bringing the total to over $40 billion.^2^

The increasing role of ESG investment has spawned a new literature analysing whether or not ESG indices outperform conventional ones (Gladish et al. 2013; Durán-Santomil et al. 2019), and affect the performance of financial companies (Junkus and Berry 2015) or the degree of market efficiency (Mynhardt et al. 2017). In general, socially responsible companies provide more transparent reporting; this implies higher costs for the collection, compilation, disclosure, publication and verification of information according to ESG criteria, and should also result in lower information asymmetry and greater market efficiency; however, this might not be the case if reporting regulations are not sufficiently stringent.

This paper aims to shed new light on these issues by comparing two sets of 12 ESG and conventional MSGI indices to establish whether or not there are differences in their stochastic behaviour, and whether their properties are the same for different groups of countries. For this purpose two different long-memory methods, specifically R/S (Rescaled Range) analysis and fractional integration, are applied to MSCI data spanning the period 2007–2020. Therefore the present study is much more comprehensive than previous ones, such as Mynhardt et al. (2017), which focused on a smaller subset of indices and only carried out R/S analysis. Evidence of greater efficiency of the ESG indices would provide an additional reason for socially responsible investing, whilst a higher degree of predictability would provide opportunities to market participants to make abnormal profits by means of appropriately designed trading strategies.

The layout of the paper is the following. Section 2 provides a brief review of the relevant literature. Section 3 describes the data and outlines the empirical methodology. Section 4 presents the empirical results. Section 5 provides some concluding remarks.

## Literature review

The PRI (Principles for Responsible Investment), which is a UN-supported network of investors whose aim is to promote sustainable investment, was the first to define ESG criteria on the basis of which a total score is calculated for each company, which reflects the level of corporate social responsibility (CSR) and determines the weight of the company in the ESG index. ESG data are used to compare the performance of conventional versus socially responsible indices and mutual funds. Statman (2000) found that ESG indices outperform conventional ones such as the S&P 500. Cortez et al. (2009) showed that they perform better in the European markets than in the US ones. Lopez et al. (2007) compared the financial performance of companies with social-responsible investment (SRI) with that of traditional ones and found differences in the Dow Jones Sustainability Indices (DJSI) and Dow Jones Global Indices (DJGI) dynamics due to these companies’ CSR practices.

Durán-Santomil et al. (2019) reported that mutual funds investing in companies with higher ESG scores have a better performance, whilst Managi et al. (2012) and Gladish et al. (2013) found no evidence that they outperform their conventional peers. Leite and Cortez (2013) confirmed that differences between SRI funds and conventional ones are not statistically significant.

El Ghoul and Karoui (2016) concluded that high-CSR funds are outperformed by low-CSR ones as their investors derive utility from non-performance attributes. Cortez and Leite (2015) argued that in general ESG indices underperform during normal periods, whilst during turmoil periods such as the 2007 global financial crisis (GFC) they outperform conventional ones because they play an ‘insurance role’ (Varma and Nofsinger 2014; Becchetti et al. 2015). Abidin and Gan (2017), Junkus and Berry (2015), Rehman et al. (2016) and Schröder (2004) showed that the performance of SRI mutual funds and indices is generally not significantly different from that of conventional ones. Rehman et al. (2021) reported that in the case of the BRICS (Brazil, Russia, India, China and South Africa) countries ESG and conventional indices influence each other. Jain et al. (2019) argued that sustainable indices and conventional ones are substitutes. As for the effects of the COVID-19 pandemic, no statistically significant differences have been detected for the returns of ESG indices compared to traditional ones (Chiappini et al. 2021; Umar et al. 2020).

The mixed results of the studies discussed above can be attributed to differences in model specifications, sample periods, benchmarks etc. (Junkus and Berry 2015). The heterogeneity of sustainable investment in terms of its performance provides an opportunity to reduce risk by diversifying across regions (Cunha et al. 2020); this type of investment is not necessarily penalising for investors who could switch to it without incurring losses (Tripathi and Kaur 2020; Sharma et al. 2021).

Very few studies focus on the issue of persistence of ESG indices vis-a-vis conventional ones. In particular, Mynhardt et al. (2017) examined the persistence of the DJSI, S&P500 Environmental & Socially Responsible Index, FTSE4 Good Global Index, MSCI World ESG Index, NASDAQ OMX CRD Global Sustainability Index, and their traditional equivalents with R/S analysis; they found that generally SRI indices exhibit lower efficiency than traditional ones. The only previous study to use fractional integration techniques is due to de Dios-Alija et al. (2021), who analysed the Dow Jones, Eurostoxx, and Hang Seng monthly and weekly sustainable and traditional indices; high levels of persistence were observed in all cases and no differences were detected across markets. Persistence is a measure of market efficiency as discussed by Mandelbrot (1972) and Peters (1991, 1994). Previous studies analysing it for various financial markets also include Greene and Fielitz (1977), Lo (1991), Jacobsen (1995), Costa and Vasconcelos (2003), Onali and Goddard (2011), Caporale et al. (2016).

## Data and methodology

We analyse two sets of 12 ESG and conventional daily indices from the MSCI (Morgan Stanley Capital International) website https://www.msci.com/. The sample period goes from 1 October 2007 to 31 December 2020 (with the only exception of the MSCI BRIC ESG series which starts on 12 July 2013). Specifically, the following (both ESG and conventional) MSCI indices are examined: US, UK, Japan, India, China, South Africa, Emerging Markets (including 27 emerging markets such as Argentina, Brazil, Egypt, Malaysia, Mexico, etc.), BRICS (Brazil, Russia, India, China, South Africa), World (including 23 developed markets, such as the US, Japan, UK, France, etc.), Europe (including 15 European developed markets such as Germany, Italy, Netherlands, the UK, etc.), Pacific (including 5 developed markets in the Pacific region, specifically Japan, Hong Kong, Australia, Singapore, and New Zealand), EAFE (a broad market index of stocks from Europe, Australasia, and the Middle East which includes more than 900 stocks from 21 countries).

To measure the degree of persistence of these series two different methods are applied, namely R/S (Rescaled Range) analysis and fractional integration methods. The former is based on the Hurst exponent *H*, which is the estimated slope coefficient in the following equation: *log (R / S)* = *log (c)* + H*log (n) (Hurst 1951). For each sub-period the range R (the difference between the maximum and minimum value of the index within the sub-period), the standard deviation S and their average ratio R/S are calculated. The length of the sub-period is increased and the calculations repeated until the sub-period coincides with the full sample. Each sub-period is characterised by its average value of R/S. The least square method is applied to these values and a regression is run, obtaining an estimate of the slope coefficient, which, as already mentioned, is known as the Hurst exponent. More details are provided below.

1. One starts with a time series of length *M* and transforms it into one of length *N* = *M—1* using logs and converting stock prices into stock returns:
  1 $${N}_{i}=\mathit{log}\left(\frac{{Y}_{t+1}}{{Y}_{t}}\right),t=\mathrm{1,2},3,...(M-1)$$
2. One divides this period into contiguous sub-periods *A* with length n, such that *A*_*n*_ = *N*, then one identifies each sub-period as *I*_*a*_ for *a* = 1, 2, 3..., *A*. Each element of *I*_*a*_ is denoted by *N*_*k*_, with *k* = 1, 2, 3..., *N*. For each *I*_*a*_ the average sub-period return ${e}_{a}$ is defined as:
  2 $${e}_{a}=\frac{1}{n}{\sum }_{k=1}^{n}{N}_{k,a}$$
3. The accumulated deviations *X*_*k,a*_ from the average ${e}_{a}$ for each sub-period *I*_*a*_ are defined as:
  3 $${X}_{k,a}=\sum_{i=1}^{k}({N}_{i,a}-{e}_{a}).$$
  The range is defined as the maximum *X*_*k,a*_ minus the minimum *X*_*k,a*_, within each sub-period (*I*_*a*_):
  4 $${R}_{Ia}=\mathrm{max}({X}_{k,a})\text{\hspace{0.17em}\hspace{0.17em}}-\text{\hspace{0.17em}\hspace{0.17em}}\mathrm{min}({X}_{k,a}),\text{\hspace{0.17em}\hspace{0.17em}\hspace{0.17em}\hspace{0.17em}\hspace{0.17em}}1\text{\hspace{0.17em}\hspace{0.17em}}\le \text{\hspace{0.17em}\hspace{0.17em}}k\text{\hspace{0.17em}\hspace{0.17em}}\le \text{\hspace{0.17em}\hspace{0.17em}\hspace{0.17em}}n.$$
4. The standard deviation ${S}_{Ia}$ is calculated for each sub-period *I*_*a*_:
  5 $${S}_{Ia}\text{\hspace{0.17em}\hspace{0.17em}}=\text{\hspace{0.17em}\hspace{0.17em}\hspace{0.17em}\hspace{0.17em}}{\left((\frac{1}{n})\sum_{k=1}^{n}{({N}_{k,a}-\text{\hspace{0.17em}\hspace{0.17em}}{e}_{a})}^{2}\right)}^{\mathrm{0,5}}.$$
5. Each range *R*_*Ia*_ is normalised by dividing by the corresponding *S*_*Ia*_. Therefore, the re-normalised scale during each sub-period *I*_*a*_ is *R*_*Ia*_*/ S*_*Ia*_. In the step 2 above, one obtains adjacent sub-periods of length n. Thus, the average R/S for length *n* is defined as:
  6 $${(R/S)}_{n}\text{\hspace{0.17em}\hspace{0.17em}}=\text{\hspace{0.17em}\hspace{0.17em}\hspace{0.17em}}(1/A)\sum_{i=1}^{A}({R}_{Ia}/{S}_{Ia}).$$
6. The length *n* is increased to the next higher level, *(M—1)/n*, and must be an integer number. In our case, we use *n*-indices that include the initial and end points of the time series, and Steps 1—6 are repeated until *n* = *(M—1)/2*.
7. Next one can use least square to estimate the equation *log (R / S)* = *log (c)* + *Hlog (n).* The slope coefficient in this regression is an estimate of the Hurst exponent *H*. To assess the statistical significance of the estimated Hurst exponent coefficients p-values and 95% confidence intervals can also be computed in the standard way in the context of regression analysis. Note that the Hurst exponent lies in the interval [0, 1]. On the basis of the H values three categories can be identified: the series are anti-persistent, and returns are negatively correlated if 0 ≤ H < 0.5; the series are random, returns are uncorrelated, and there is no memory in the series if H = 0.5; the series are persistent, returns are highly correlated, and there is memory in price dynamics if 0.5 < H ≤ 1.

Both static and dynamic R/S analysis is carried out. In the former case the Hurst exponent is calculated using the whole data set. In the latter a sliding-window approach is used and a series of Hurst exponents corresponding to each window are obtained. The procedure is the following: having computed the first value of the Hurst exponent (for example, for the date 01.04.2004 using data for the period from 01.01.2004 to 31.03.2004), each of the following ones is calculated by shifting forward the ‘data window’, where the size of the shift depends on the number of observations and a sufficient number of estimates (namely > 100 following the literature) is required to analyse the time-varying behaviour of the Hurst exponent. For example, if the shift equals 10, the second value is calculated for 10.04.2004 and characterises the market over the period 10.01.2004 till 09.04.2004, and so on.

The second method employs fractional integration techniques to estimate the differencing parameter d as a measure of persistence; note that this is related to the Hurst exponential described above through the relationship H = d + 0.5. Further, R/S analysis is applied to the return series (the first differences of the logged indices), while I(d) models are estimated for the logged indices themselves, in which case the relationship becomes H = (d – 1) + 0.5 = d – 0.5. We consider processes of the form:

7 $${(1\text{\hspace{0.17em}\hspace{0.17em}\hspace{0.17em}}-\text{\hspace{0.17em}\hspace{0.17em}}B)}^{d}{x}_{t}\text{\hspace{0.17em}\hspace{0.17em}\hspace{0.17em}\hspace{0.17em}}=\text{\hspace{0.17em}\hspace{0.17em}\hspace{0.17em}}{u}_{t}\text{\hspace{0.17em}},\text{\hspace{0.17em}\hspace{0.17em}\hspace{0.17em}\hspace{0.17em}\hspace{0.17em}\hspace{0.17em}\hspace{0.17em}\hspace{0.17em}\hspace{0.17em}\hspace{0.17em}\hspace{0.17em}\hspace{0.17em}\hspace{0.17em}\hspace{0.17em}\hspace{0.17em}\hspace{0.17em}\hspace{0.17em}\hspace{0.17em}\hspace{0.17em}\hspace{0.17em}}t\text{\hspace{0.17em}\hspace{0.17em}\hspace{0.17em}\hspace{0.17em}}=\text{\hspace{0.17em}\hspace{0.17em}\hspace{0.17em}\hspace{0.17em}}1\text{\hspace{0.17em}},\text{\hspace{0.17em}\hspace{0.17em}\hspace{0.17em}}2\text{\hspace{0.17em}},\text{\hspace{0.17em}\hspace{0.17em}}...\text{\hspace{0.17em}},$$

where B is the backshift operator (Bx_t_ = x_t-1_); u_t_ is an I(0) process (which may incorporate weak autocorrelation of the AR(MA) form) and x_t_ represents the errors of a regression model of the form:

8 $${\text{y}}_{\text{t}}\text{\hspace{0.17em}\hspace{0.17em}\hspace{0.17em}}=\text{\hspace{0.17em}\hspace{0.17em}\hspace{0.17em}\hspace{0.17em}}{\beta }_{0}\text{\hspace{0.17em}\hspace{0.17em}\hspace{0.17em}}+\text{\hspace{0.17em}\hspace{0.17em}\hspace{0.17em}}{\beta }_{1}\text{\hspace{0.17em}}t\text{\hspace{0.17em}\hspace{0.17em}}+{x}_{\text{t}};\text{\hspace{0.17em}\hspace{0.17em}\hspace{0.17em}\hspace{0.17em}\hspace{0.17em}\hspace{0.17em}\hspace{0.17em}\hspace{0.17em}\hspace{0.17em}\hspace{0.17em}\hspace{0.17em}\hspace{0.17em}\hspace{0.17em}\hspace{0.17em}\hspace{0.17em}\hspace{0.17em}\hspace{0.17em}}t\text{\hspace{0.17em}\hspace{0.17em}\hspace{0.17em}\hspace{0.17em}}=\text{\hspace{0.17em}\hspace{0.17em}\hspace{0.17em}\hspace{0.17em}}1\text{\hspace{0.17em}},\text{\hspace{0.17em}\hspace{0.17em}\hspace{0.17em}}2\text{\hspace{0.17em}},\text{\hspace{0.17em}\hspace{0.17em}}...\text{\hspace{0.17em}},$$

where y_t_ stands for the log of the stock index in each case, β_0_ and β_1_ denote an unknown constant and coefficient on a linear time trend t, and the regression errors x_t_ are I(d). Note that under the Efficient Market Hypothesis the value of d in (7) should be equal to 1 and u_t_ should be a white noise process. We use parametric and semiparametric methods, assuming in turn uncorrelated (white noise) and autocorrelated errors as in Bloomfield (1973). To be more specific, in the former case the model including Eqs. (7) and (8) is fully parameterised, with the assumption being made that u_t_ in (7) is a white noise process; in the latter no structure is imposed on u_t_, which is allowed to be weakly autocorrelated as in Bloomfield (1973)—his model can be characterised as semiparametric because it does not have an explicit specification but can be described simply by its spectral density function, whose logged form approximates that of autoregressive models. In both cases, we use the Whittle estimator of d in the frequency domain (Dahlhaus 1989; Robinson 1994, 1995), as described, for example, in Gil-Alana and Robinson (1997).

## Empirical results

The results of the static R/S analysis for the ESG and conventional MSCI indices are reported in Table 1.

**Table 1:** Static Hurst exponent calculations for the ESG and conventional MSCI indices (R/S analysis)

Index	ESG (*p*-values and confidence intervals—CI*)	Conventional (*p*-values and confidence intervals—CI)	Difference, %
MSCI USA	0.53 (p = 0.00; CI = 0.51–0.54)	0.53 (p = 0.00; CI = 0,51–0,55)	0%
MSCI UK ESG	0.53 (p = 0.00; CI = 0.51–0.54)	0.53 (p = 0.00; CI = 0.51–0.54)	0%
MSCI CHINA ESG	0.57 (p = 0.00; CI = 0.56–0.58)	0.58 (p = 0.00; CI = 0.57–0.59)	-2%
MSCI INDIA ESG	0.54 (p = 0.00; CI = 0.53–0.55)	0.56 (p = 0.00; CI = 0.54–0.57)	-2%
MSCI JAPAN ESG	0.53 (p = 0.00; CI = 0.51–0.54)	0.53 (p = 0.00; CI = 0.52–0.54)	-1%
MSCI SOUTH AFRICA	0.51 (p = 0.00; CI = 0.49–0.53)	0.51 (p = 0.00; CI = 0.50–0.53)	-1%
MSCI WORLD	0.55 (p = 0.00; CI = 0.53–0.57)	0.56 (p = 0.00; CI = 0.54–0.57)	-1%
MSCI BRIC	0.60 (p = 0.00; CI = 0.59–0.61)	0.59 (p = 0.00; CI = 0.58–0.60)	2%
MSCI EMERGING MKTS	0.58 (p = 0.00; CI = 0.57–0.59)	0.59 (p = 0.00; CI = 0.58–0.60)	-1%
MSCI EAFE	0.56 (p = 0.00; CI = 0.54–0.57)	0.56 (p = 0.00; CI = 0.54–0.57)	-1%
MSCI EUROPE	0.54 (p = 0.00; CI = 0.53–0.55)	0.54 (p = 0.00; CI = 0.53–0.55)	0%
MSCI PACIFIC	0.54 (p = 0.00; CI = 0.53–0.56)	0.55 (p = 0.00; CI = 0.54–0.56)	-1%

*p-values are reported concerning the statistical significance of the estimated Hurst exponents

As can be seen, all calculated p-values are below 0.05, which implies that the estimated Hurst exponents are statistically significant, and in most cases there are no significant differences between the two types of indices; it is noteworthy that the estimates are generally higher for the emerging markets considered, which suggests that these are less efficient than the developed ones (in line with previous evidence)—the general consensus is that this is due to the fact that such markets are generally characterised by greater information asymmetry, lower liquidity, and fewer market participants.

**Fig. 1:**
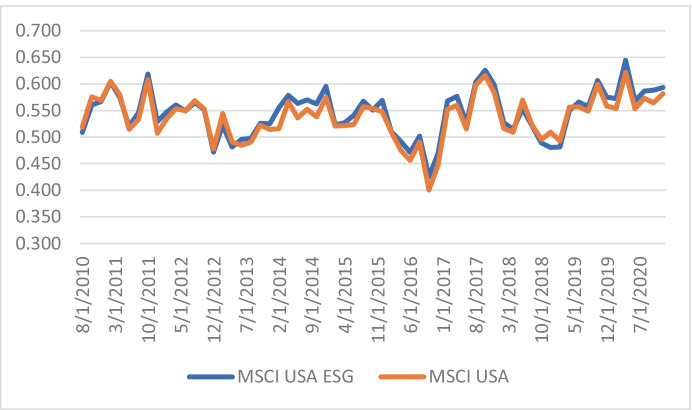
Dynamic R/S analysis of the ESG and conventional MSCI indices: the case of MSCI USA

**Fig. 2:**
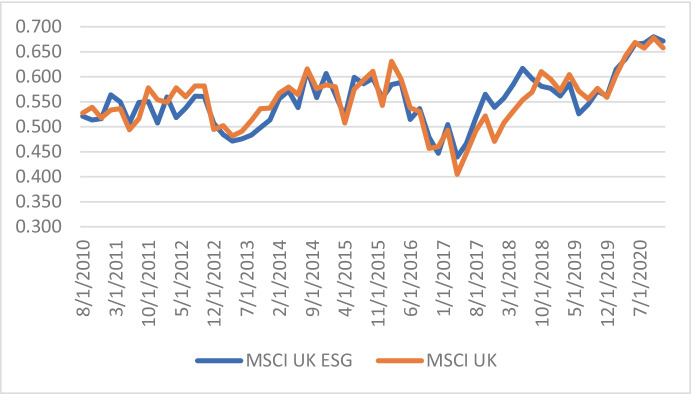
Dynamic R/S analysis of the ESG and conventional MSCI indices: the case of MSCI UK

**Fig. 3:**
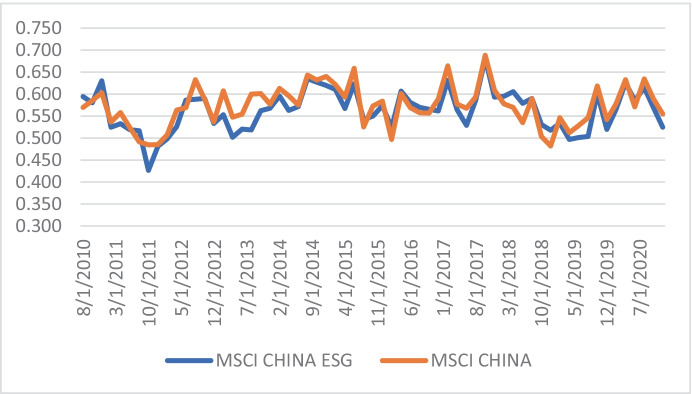
Dynamic R/S analysis of the ESG and conventional MSCI indices: the case of MSCI China

**Fig. 4:**
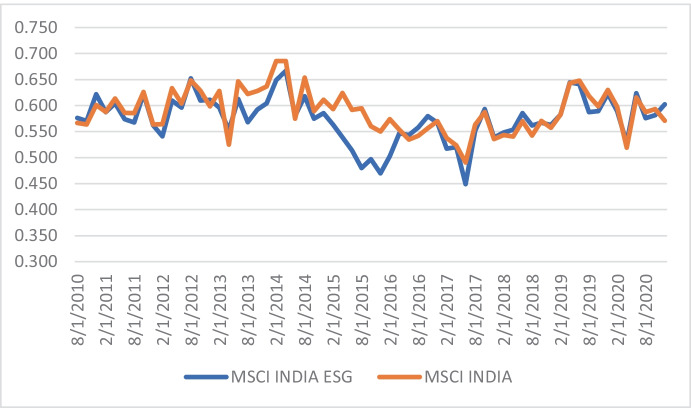
Dynamic R/S analysis of the ESG and conventional MSCI indices: the case of MSCI India

**Fig. 5:**
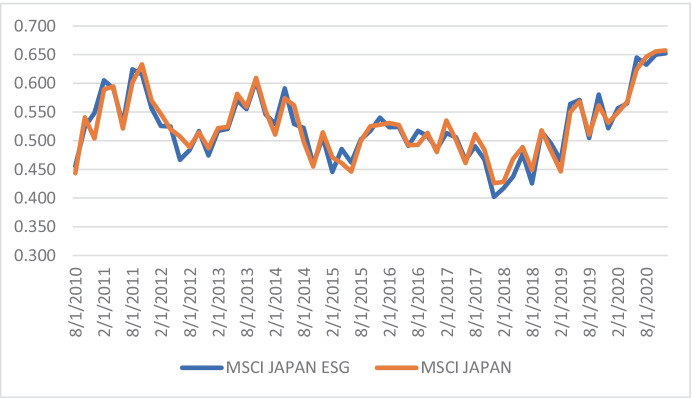
Dynamic R/S analysis of the ESG and conventional MSCI indices: the case of MSCI Japan

**Fig. 6:**
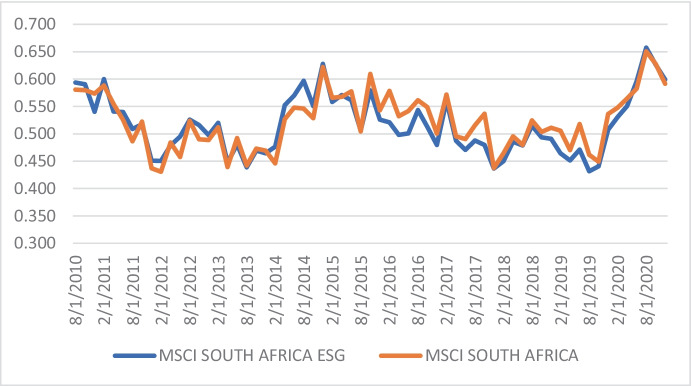
Dynamic R/S analysis of the ESG and conventional MSCI indices: the case of MSCI South Africa

**Fig. 7:**
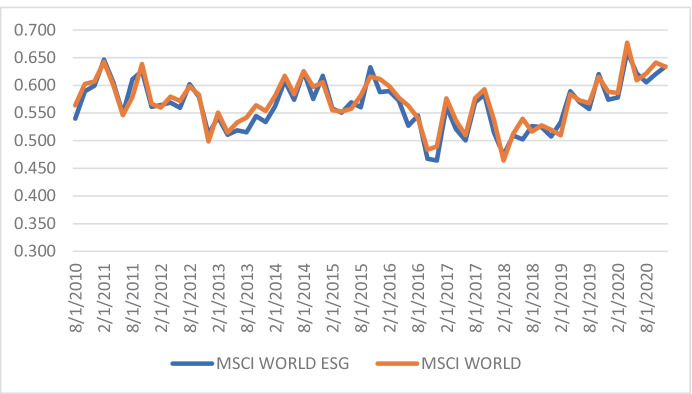
Dynamic R/S analysis of the ESG and conventional MSCI indices: the case of MSCI World

**Fig. 8:**
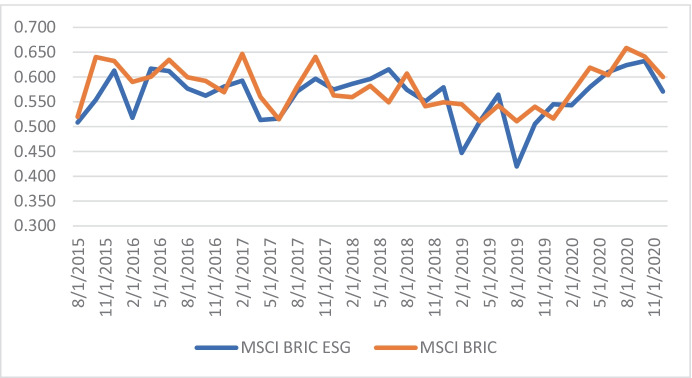
Dynamic R/S analysis of the ESG and conventional MSCI indices: the case of MSCI BRIC

**Fig. 9:**
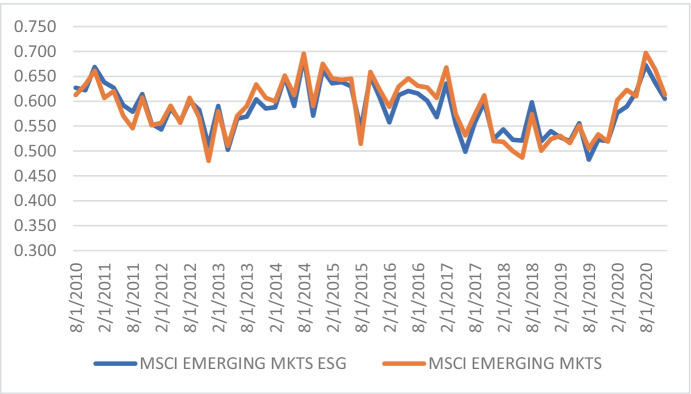
Dynamic R/S analysis of the ESG and conventional MSCI indices: the case of MSCI Emerging Markets

**Fig. 10:**
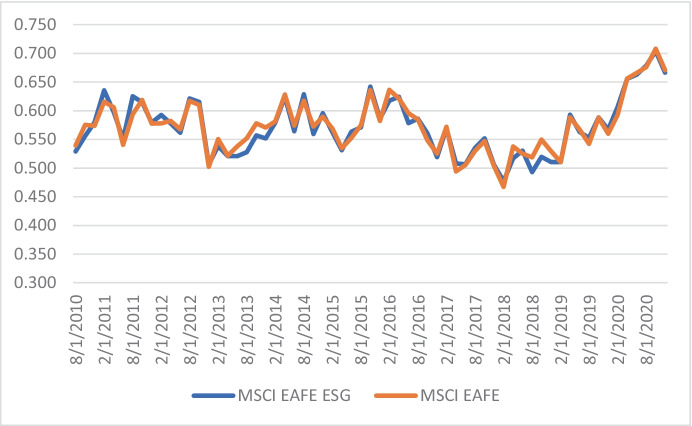
Dynamic R/S analysis of the ESG and conventional MSCI indices: the case of MSCI EAFE

**Fig. 11:**
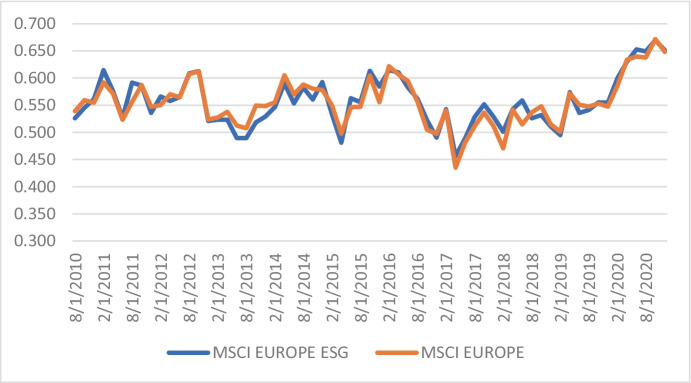
Dynamic R/S analysis of the ESG and conventional MSCI indices: the case of MSCI Europe

**Fig. 12:**
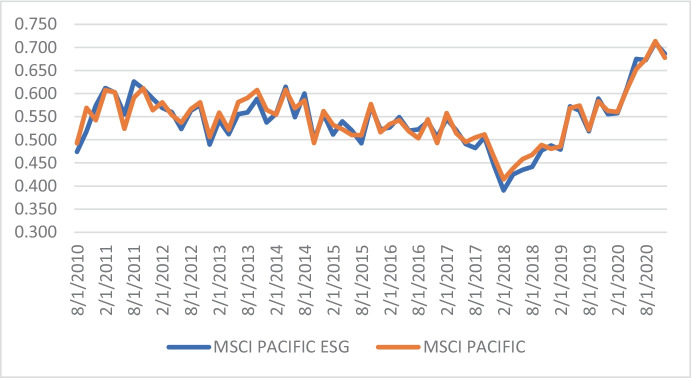
Dynamic R/S analysis of the ESG and conventional MSCI indices: the case of MSCI BRIC

The next step is the dynamic R/S analysis, which provides information about changes in persistence over time. The results are plotted in Appendix 1, Figures 1, 2, 3, 4, 5, 6, 7, 8, 9, 10, 11 and 12. Visual inspection suggests that persistence is time-varying and that its dynamic behaviour is very similar for the ESG and conventional indices. This is confirmed by the results reported in Table 2—with very few exceptions (the BRICS and India by itself) the correlations between the Hurst exponents obtained for the two types of indices are very high.

**Table 2:** Correlation analysis of Hurst exponent dynamics for the ESG and conventional indices

Country/Region	Correlation coefficient
USA	0.96
UK	0.89
CHINA	0.85
INDIA	0.77
JAPAN	0.96
SOUTH AFRICA	0.91
WORLD	0.96
BRICS	0.68
EMERGING MKTS	0.94
EAFE	0.97
EUROPE	0.95
PACIFIC	0.97

As an additional check we also carry out t-tests to see whether or not there are any statistically significant differences between the ESG and conventional indices in terms of Hurst exponent dynamics. The results are presented in Appendix 2 (Tables 9, 10 and 11). The null hypothesis of no difference is rejected only in the case of India. To sum up, the R/S analysis implies that persistence and its dynamics are essentially the same for the two sets of indices. However, persistence tends to be higher in emerging as opposed to developed markets, which indicates that the former are less efficient, a common finding in the literature.

More evidence is obtained using I(d) techniques. Specifically, we estimate the model given by Eqs. (7) and (8) and report the results for the two cases of white noise and autocorrelated errors in Table 3 and 4 for the ESG indices and in Tables 5 and 6 for the conventional ones; in particular, these tables display the estimates of d as well as their 95% confidence intervals obtained as the values of d_o_ for which the null hypothesis of d = d_o_ cannot be rejected at the 5% level with the tests of Robinson (1994). In each case we display the estimates of d for three standard model specifications, namely: i) no deterministic terms (i.e., β_0_ = β_1_ = 0 in (8)), ii) an intercept only (β_1_ = 0), and iii) an intercept and a linear time trend. The values in bold are those from the preferred specifications selected on the basis of the statistical significance of the regressors.

**Table 3:** Estimates of d based on white noise errors – ESG indices

Series	No deterministic terms	An intercept	An intercept and a linear time trend
USA	-0.086 (-0.103, -0.068)	-0.087 (-0.105, -0.067)	**-0.089 ( +)** **(-0.108, -0.070)**
UK	**-0.027** **(-0.049, -0.002)**	-0.027 (-0.049, -0.002)	-0.027 (-0.049, -0.002)
CHINA	-0.018 (-0.048, 0.006)	-0.018 (-0.048, 0.006)	**-0.020 ( +)** **(-0.052, 0.004)**
INDIA	**0.009** **(-0.013, 0.033)**	0.009 (-0.013, 0.033)	0.009 (-0.014, 0.032)
JAPAN	-0.099 (-0.118, -0.079)	-0.099 (-0.118, -0.079)	**-0.103 ( +)** **(-0.120, -0.081)**
SOUTH AFRICA	**0.001** **(-0.024, -0.028)**	0.001 (-0.023, -0.028)	0.001 (-0.024, -0.028)
WORLD	**0.029** **(0.006, 0.054)**	0.029 (0.006, 0.054)	0.028 (0.005, 0.053)
EMERGING MKTS	**0.109** **(0.083, 0.139)**	0.109 (0.083, 0.139)	0.109 (0.083, 0.138)
EAFE	**0.057** **(0.032, 0.084)**	0.057 (0.032, 0.084)	0.056 (0.031, 0.083)
EUROPE	**-0.016** **(-0.038, 0.007)**	-0.016 (-0.038, 0.007)	-0.018 (-0.039, 0.006)
PACIFIC	**-0.034** **(-0.055, -0.012)**	-0.034 (-0.055, -0.012)	-0.035 (-0.056, -0.012)
BRICS	**0.047** **(-0.016, 0.082)**	0.047 (-0.016, 0.082)	0.046 (-0.015, 0.081)

The values in bold are those from the specification selected on the basis of the statistical significance of the deterministic terms; in brackets the corresponding confidence intervals.

**Table 4:** Estimates of d based on autocorrelated errors – ESG indices

Series	No deterministic terms	An intercept	An intercept and a linear time trend
USA	0.008 (-0.023, 0.054)	0.008 (-0.023, 0.054)	**0.008** **(-0.023, 0.054)**
UK	**-0.082** **(-0.122, -0.033)**	-0.082 (-0.122, -0.033)	-0.083 (-0.123, -0.032)
CHINA	**-0.043** **(-0.088, -0.001)**	-0.044 (-0.089, -0.001)	-0.051 (-0.088, -0.014)
INDIA	**0.031** **(-0.009, 0.060)**	0.031 (-0.009, 0.060)	0.031 (-0.010, 0.060)
JAPAN	**-0.059** **(-0.092, -0.011)**	-0.059 (-0.092, -0.012)	-0.059 (-0.093, -0.012)
SOUTH AFRICA	**-0.067** **(-0.107, -0.014)**	-0.067 (-0.107, -0.014)	-0.067 (-0.108, -0.015)
WORLD	**0.011** **(-0.036, 0.063)**	0.011 (-0.036, 0.065)	0.011 (-0.036, 0.065)
EMERGING MKTS	**0.003** **(-0.042, 0.028)**	0.003 (-0.042, 0.028)	0.003 (-0.050, 0.029)
EAFE	**-0.037** **(-0.037, 0.022)**	-0.037 (-0.037, 0.022)	-0.037 (-0.074, 0.023)
EUROPE	**-0.058** **(-0.087, -0.021)**	-0.058 (-0.087, -0.021)	-0.054 (-0.087, -0.021)
PACIFIC	**-0.019** **(-0.055, 0.032)**	-0.019 (-0.054, 0.032)	-0.019 (-0.055, 0.033)
BRICS	0.008 (-0.044, 0.061)	0.008 (-0.044, 0.061)	**0.007 ( +)** **(-0.045, 0.058)**

The values in bold are those from the specification selected on the basis of the statistical significance of the deterministic terms; in brackets the corresponding confidence intervals

**Table 5:** Estimates of d based on white noise errors—conventional indices

Series	No deterministic terms	An intercept	An intercept and a linear time trend
USA	-0.084 (-0.107, -0.069)	-0.085 (-0.108, -0.068)	**-0.089** **(-0.108, -0.067)**
UK	**-0.024** **(-0.041, -0.002)**	-0.024 (-0.042, -0.002)	-0.024 (-0.043, -0.001)
CHINA	**0.007** **(-0.016, 0.034)**	0.007 (-0.016, 0.034)	0.005 (-0.018, 0.034)
INDIA	**0.033** **(0.012, 0.052)**	0.033 (0.012, 0.052)	0.033 (0.012, 0.051)
JAPAN	-0.097 (-0.114, -0.074)	-0.097 (-0.114, -0.074)	**-0.100** **(-0.114, -0.079)**
SOUTH AFRICA	**0.001** **(-0024, 0.027)**	0.001 (-0024, 0.027)	0.001 (-0024, 0.027)
WORLD	**0.030** **(0.006, 0.057)**	0.030 (0.007, 0.058)	0.029 (0.007, 0.060)
EMERGING MKTS	**0.126** **(0.094, 0.156)**	0.126 (0.094, 0.157)	0.126 (0.095, 0.156)
EAFE	**0.063** **(0.031. 0.089)**	0.063 (0.031. 0.089)	0.062 (0.032, 0.089)
EUROPE	**-0.013** **(-0.034, 0.011)**	-0.013 (-0.034, 0.012)	-0.014 (-0.034, 0.012)
PACIFIC	**-0.021** **(-0.046, -0.004)**	-0.021 (-0.045, -0.004)	-0.021 (-0.045, -0.005)
BRICS	**0.097** **(0.071, 0.122)**	0.097 (0.072, 0.122)	0.097 (0.072, 0.123)

The values in bold are those from the specification selected on the basis of the statistical significance of the deterministic terms; in brackets the corresponding confidence intervals

**Table 6:** Estimates of d based on autocorrelated errors—conventional indices

Series	No deterministic terms	An intercept	An intercept and a liner time trend
USA	**-0.039** **(-0.090, 0.004)**	-0.042 (-0.091, 0.004)	-0.048 (-0.091, 0.004)
UK	**-0.075** **(-0.110, -0.041)**	-0.075 (-0.109, -0.041)	-0.076 (-0.112, -0.042)
CHINA	**-0.028** **(-0.059, -0.001)**	-0.028 (-0059, -0.001)	-0.027 (-0061, -0.001)
INDIA	**-0.006** **(-0.031, 0.036)**	-0.006 (-0.031, 0.036)	-0.005 (-0.031, 0.035)
JAPAN	**-0.059** **(-0.101, -0.024)**	-0.059 (-0.100, -0.024)	-0.062 (-0.101, -0.023)
SOUTH AFRICA	**-0.099** **(-0.131, -0.058)**	-0.099 (-0.131, -0.058)	-0.099 (-0.131, -0.058)
WORLD	-0.056 (-0.074, -0.018)	-0.056 (-0.080, -0.018)	**-0.059** **(-0.080, -0.021)**
EMERGING MKTS	**-0.009** **(-0.041, 0.036)**	-0.009 (-0.042, 0.035)	-0.009 (-0.042, 0.037)
EAFE	**-0.057** **(-0.081, -0.018)**	-0.057 (-0.081, -0.018)	-0.059 (-0.082, -0.019)
EUROPE	**-0.048** **(-0.093, -0.024)**	-0.048 (-0.093, -0.024)	-0.056 (-0.093, -0.013)
PACIFIC	**-0.036** **(-0.075, -0.004)**	-0.036 (-0.075, -0.004)	-0.038 (-0.075, -0.005)
BRICS	**-0.028** **(-0.054, 0.016)**	-0.028 (-0.054, 0.016)	-0.029 (-0.054, 0.016)

The values in bold are those from the specification selected on the basis of the statistical significance of the deterministic terms; in brackets the corresponding confidence intervals.

Starting with the ESG indices, under the assumption of white noise errors we find a significant time trend (with a positive coefficient, not reported) in the case of China, Japan and the US, whilst in the remaining cases neither an intercept nor a trend is required. Long memory (d > 0) characterises the BRICS, EAFE, Emerging Markets, and World indices; evidence of short memory (d = 0) is found for China, Europe, India and South Africa, while anti-persistence (d < 0) is detected for Japan, Pacific, the UK and the US.

When allowing for autocorrelation, the time trend is significant only in the case of the BRICS and China. There is no a single case of long memory; I(0) or short memory is found for the BRICS, EAFE, the Emerging Markets, India, Pacific, US and World indices, while for the remaining series (China, Europe, Japan, South Africa and the UK) d is significantly smaller than 0, which amounts to anti-persistence.

Next, we analyse the conventional indices. With white noise errors (Table 5), the time trend is significant for the US and Japan, while in the remaining cases no deterministic terms are required. As for the estimated values of d, anti-persistence (i.e. d < 0) is found in the case of the US, UK, Japan and the Pacific; evidence of short memory or I(0) behaviour is obtained for Europe, China and South Africa, and long memory (i.e., d > 0) is detected in the case of the India, World, Emerging Markets, EAFE and BRICS indices.

Under the assumption of correlated errors the time trend is only significant for the World index, whilst in the remaining cases both the intercept and the time trend are insignificant. Anti-persistence is found in the case of the UK, China, Japan, South Africa, the World, EAFE, Europe and the Pacific, and short memory (d = 0) for the US, India and the BRICS, thus long memory is not found in any single case.

Table 7 and 8 provide a synoptic view respectively of the estimates of the differencing parameter d and of the findings concerning the presence of anti-persistence (AP, i.e., a statistically significant coefficient d < 0 at the 95% level, marked with * in Table 7), short memory (SM, d = 0) and long memory (LM i.e., a statistically significantly coefficient d > 0 at the 95% level, marked with + in Table 7) on the basis of the estimated values of d.

**Table 7:** Summary of the estimates of the differencing parameter d

Method	White noise errors		Autocorrelated errors	
Countries	ESG	Conventional	ESG	Conventional
USA	-0.089^*^ (-0.108, -0.070)	-0.089^*^ (-0.108, -0.067)	0.008 (-0.023, 0.054)	-0.039 (-0.090, 0.004)
UK	-0.027^*^ (-0.049, -0.002)	-0.024^*^ (-0.041, -0.002)	-0.082^*^ (-0.122, -0.033)	-0.075^*^ (-0.110, -0.041)
CHINA	-0.020 (-0.052, 0.004)	0.007 (-0.016, 0.034)	-0.043^*^ (-0.088, -0.001)	-0.028^*^ (-0.059, -0.001)
INDIA	0.009^*^ (-0.013, 0.033)	0.033^+^ (0.012, 0.052)	0.031 (-0.009, 0.060)	-0.006 (-0.031, 0.036)
JAPAN	-0.103^*^ (-0.120, -0.081)	-0.100^*^ (-0.114, -0.079)	-0.059^*^ (-0.092, -0.011)	-0.059^*^ (-0.101, -0.024)
SOUTH AFRICA	0.001^*^ (-0.024, -0.028)	0.001 (-0024, 0.027)	-0.067^*^ (-0.107, -0.014)	-0.099^*^ (-0.131, -0.058)
WORLD	0.029^+^ (0.006, 0.054)	0.030^+^ (0.006, 0.057)	0.011 (-0.036, 0.063)	-0.059^*^ (-0.080, -0.021)
EMERGING MKTS	0.109^+^ (0.083, 0.139)	0.126^+^ (0.094, 0.156)	0.003 (-0.042, 0.028)	-0.009 (-0.041, 0.036)
EAFE	0.057^+^ (0.032, 0.084)	0.063^+^ (0.031. 0.089)	-0.037 (-0.037, 0.022)	-0.057^*^ (-0.081, -0.018)
EUROPE	-0.016 (-0.038, 0.007)	-0.013 (-0.034, 0.011)	-0.058^*^ (-0.087, -0.021)	-0.048^*^ (-0.093, -0.024)
PACIFIC	-0.034^*^ (-0.055, -0.012)	-0.021^*^ (-0.046, -0.004)	-0.019 (-0.055, 0.032)	-0.036^*^ (-0.075, -0.004)
BRICS	0.047 (-0.016, 0.082)	0.097^+^ (0.071, 0.122)	0.007 (-0.045, 0.058)	-0.028 (-0.054, 0.016)

*: Evidence of Anti-Persistence (d < 0) at the 95% level: + : Evidence of long memory (d > 0) at the 95% level

**Table 8:** Summary of the results based on the estimates of d: anti-persistence (AP), short memory (SM) and long memory (LM)

	White noise errors		Autocorrelated errors	
Countries	ESG	Conventional	ESG	Conventional
USA	AP	AP	SM	SM
UK	AP	AP	AP	AP
CHINA	SM	SM	AP	AP
INDIA	SM	LM	SM	SM
JAPAN	AP	AP	AP	AP
SOUTH AFRICA	AP	SM	AP	AP
WORLD	LM	LM	SM	AP
EMERGING MKTS	LM	LM	SM	SM
EAFE	LM	LM	SM	AP
EUROPE	SM	SM	AP	AP
PACIFIC	AP	AP	SM	AP
BRICS	SM	LM	SM	SM

As can be seen, with white noise errors, there are differences between the two sets of indices only in the case of India and the BRICS, where short memory (d = 0) characterises the ESG indices and long memory (d > 0) the conventional ones, and also in the case of South Africa, where the ESG index exhibits anti-persistence and the conventional one short memory instead. By contrast, when allowing for autocorrelation, differences are found in the case of the World, EAFE and Pacific indices, the ESG ones being characterised by short memory (d = 0) and the conventional ones by anti-persistence (d < 0).

In general, the fractional integration results confirm those based on the R/S analysis, namely there are no significant differences in terms of the degree of persistence between the two sets of indices. Further, higher persistence is found for emerging markets than for developed ones, the former appearing to be less efficient. These findings imply that trading and investment strategies based on the ESG indices are not more profitable, though there might be scope for abnormal profits in the case of the less efficient emerging markets (the BRICS in particular).

Possible explanations for these results include different types of “camouflage” or “washing” (see Gray 2006), namely the misrepresentation of a company’s ESG record by exaggerating its environmental credentials (“green washing”), overstating the impact of an investment on labour or human rights (“social washing”), creating the false impression of being LGBT (lesbian, gay, bisexual, and transgender) friendly (“pink washing”), signing up for the UN compact and using the UN logo to shift attention from controversial business practices (“blue washing”), or highlighting progress towards some Sustainable Development Goals (SDG) whilst hiding some questionable business practices in the pursuit of profit (“SDG washing”). In all such cases companies, despite their alleged ESG credentials, behave in the same way as conventional, profit-seeking ones and thus it is not surprising that the statistical properties of their stocks and the corresponding indices should be the same.

In practice it is often difficult to identify “washing” given the existing regulations on ESG reporting; for instance, only on 10 March 2021 was the EU Regulation 2019/2088 proposed by the European Council on 27 November 2019 approved by the European Parliament; this is an attempt to create a classification of green (sustainable) activities and regulate their disclosure. It is noteworthy that the BRICs countries are leaders in implementing ESG reporting practices. In 2020, they were among the top 20 countries in terms of ESG reporting regulations and the share of companies reporting on sustainability (India: 18 regulations, 98% of reporting companies; Brazil: 18 and 85% respectively; China: 15 and 78% respectively—KPMG 2020; Van der Lugt et al. 2020). For example, in India, all listed companies are required to disclose sustainability information in annual reports; Brazil has introduced 'report or explain' requirements related to the SDGs, and in China even state-owned companies disclose information on ESG criteria and SDGs (13^th^ Five Year Plan—Van der Lugt et al. 2020).

## Conclusions

This paper uses R/S analysis and fractional integration techniques to examine the persistence of two sets of 12 ESG and conventional stock price indices from the MSCI database over the period 2007–2020 for a large number of both developed and emerging markets. As ESG indices include companies with higher transparency in their case one would expect lower information asymmetry and thus higher market efficiency compared to the case of standard stock indices.

The R/S results imply that there are no significant differences between the two types of indices in terms of the degree of persistence and its dynamic behaviour. However, higher persistence is found for the emerging markets examined (especially the BRICS), which are less efficient and thus offer more opportunities for profitable trading strategies. The fractional integration analysis yields the same conclusions, namely with a few exceptions the two sets of indices exhibit very similar behaviour.

These findings can be rationalised by noting that, in the absence of stringent reporting regulations, several companies simply pretend to comply with ESG criteria while in actual fact their investment decisions are not affected by those (a phenomenon which is known as “washing” in its various forms); thus it is not surprising that their stocks should have the same persistence properties as those of conventional ones.

## Appendix 1

**Dynamic R/S analysis**

## Appendix 2

**Dynamic R/S analysis: descriptive statistic and t-tests**

**Table 9:** Descriptive statistics and t-tests of the dynamic R/S analysis results: the case of developed markets

Parameter/Index	MSCI USA		MSCI UK		MSCI JAPAN ESG		MSCI EUROPE	
	ESG	Conv	ESG	Conv	ESG	Conv	ESG	Conv
Average	0,5468	0,5391	0,5546	0,5554	0,5266	0,5283	0,5588	0,5581
Standard error	0,0055	0,0054	0,0069	0,0072	0,0076	0,0073	0,0060	0,0059
Median	0,5525	0,5481	0,5574	0,5575	0,5213	0,5207	0,5550	0,5509
Standard deviation	0,0441	0,0428	0,0553	0,0575	0,0605	0,0588	0,0478	0,0470
Sample variance	0,0019	0,0018	0,0031	0,0033	0,0037	0,0035	0,0023	0,0022
Excess	-0,0837	0,7367	0,1508	0,1808	0,0636	0,2921	-0,0095	0,5369
Asymmetry	-0,2613	-0,4970	0,3746	0,0565	0,4736	0,6593	0,4505	0,3317
Interval	0,2191	0,2207	0,2495	0,2847	0,2827	0,2682	0,2215	0,2459
Minimum	0,4254	0,4006	0,4393	0,4050	0,4024	0,4263	0,4566	0,4355
Maximum	0,6445	0,6214	0,6889	0,6897	0,6851	0,6945	0,6781	0,6814
Sum	34,9951	34,5056	35,4920	35,5448	33,7029	33,8137	35,7644	35,7172
Number of observations	64	64	64	64	64	64	64	64
t-test	0,99		-0,08		-0,16		0,09	
Null Hypothesis	not rejected		not rejected		not rejected		not rejected	

**Table 10:** Descriptive statistics and t-tests of the dynamic R/S analysis results: the case of emerging markets

Parameter/Index	MSCI CHINA		MSCI INDIA		MSCI SOUTH AFRICA		MSCI EMERGING MKTS	
	ESG	Conv	ESG	Conv	ESG	Conv	ESG	Conv
Average	0,5631	0,5744	0,5750	0,5888	0,5179	0,5235	0,5841	0,5893
Standard error	0,0057	0,0057	0,0054	0,0051	0,0067	0,0065	0,0060	0,0068
Median	0,5666	0,5763	0,5781	0,5878	0,5109	0,5242	0,5886	0,6009
Standard deviation	0,0456	0,0458	0,0436	0,0408	0,0538	0,0518	0,0476	0,0543
Sample variance	0,0021	0,0021	0,0019	0,0017	0,0029	0,0027	0,0023	0,0030
Excess	0,3171	-0,1208	0,6172	-0,1961	-0,4694	-0,5309	-0,6529	-0,8068
Asymmetry	-0,1740	-0,0127	-0,5288	0,1453	0,4805	0,1547	-0,0265	-0,1684
Interval	0,2545	0,2062	0,2178	0,1953	0,2261	0,2200	0,2061	0,2164
Minimum	0,4263	0,4823	0,4491	0,4906	0,4314	0,4306	0,4828	0,4803
Maximum	0,6808	0,6884	0,6669	0,6859	0,6575	0,6506	0,6889	0,6967
Sum	36,0390	36,7588	36,8003	37,6846	33,1468	33,5011	37,3802	37,7138
Number of observations	64	64	64	64	64	64	64	64
t-test	-1,39		-1,85		-0,59		-0,58	
Null Hypothesis	not rejected		rejected		not rejected		not rejected	

**Table 11:** Descriptive statistics and t-tests of the dynamic R/S analysis results: the case of world regions

Parameter/Index	MSCI USA		MSCI UK		MSCI JAPAN ESG		MSCI EUROPE	
	ESG	Conv	ESG	Conv	ESG	Conv	ESG	Conv
Average	0,5468	0,5391	0,5546	0,5554	0,5266	0,5283	0,5588	0,5581
Standard error	0,0055	0,0054	0,0069	0,0072	0,0076	0,0073	0,0060	0,0059
Median	0,5525	0,5481	0,5574	0,5575	0,5213	0,5207	0,5550	0,5509
Standard deviation	0,0441	0,0428	0,0553	0,0575	0,0605	0,0588	0,0478	0,0470
Sample variance	0,0019	0,0018	0,0031	0,0033	0,0037	0,0035	0,0023	0,0022
Excess	-0,0837	0,7367	0,1508	0,1808	0,0636	0,2921	-0,0095	0,5369
Asymmetry	-0,2613	-0,4970	0,3746	0,0565	0,4736	0,6593	0,4505	0,3317
Interval	0,2191	0,2207	0,2495	0,2847	0,2827	0,2682	0,2215	0,2459
Minimum	0,4254	0,4006	0,4393	0,4050	0,4024	0,4263	0,4566	0,4355
Maximum	0,6445	0,6214	0,6889	0,6897	0,6851	0,6945	0,6781	0,6814
Sum	34,9951	34,5056	35,4920	35,5448	33,7029	33,8137	35,7644	35,7172
Number of observations	64	64	64	64	64	64	64	64
t-test	0,99		-0,08		-0,16		0,09	
Null Hypothesis	not rejected		not rejected		not rejected		not rejected	

## Footnotes

1 MSCI 2021 Global Institutional Investor survey, https://www.msci.com/zh/our-clients/asset-owners/investment-insights-report

2 See https://www.cnbc.com/2020/09/19/esg-sees-record-inflows-in-2020-top-issuer-talks-staying-power.html

## References

[CR1] Abidin SZ, Gan C (2017). Do socially responsible investments strategies significantly reduce diversification benefits? Paper presented at the Proceedings - 22nd International Congress on Modelling and Simulation. MODSIM.

[CR2] Becchetti L, Ciciretti R, Dalò A, Herzel S (2015). Socially responsible and conventional investment funds: performance comparison and the global financial crisis. Appl Econ.

[CR3] Bloomfield P (1973). An exponential model for the spectrum of a scalar time series. Biometrika.

[CR4] Caporale GM, Gil-Alana L, Plastun A, Makarenko I (2016). Long memory in the Ukrainian stock market and financial crises. J Econ Finance.

[CR5] Chiappini H, Vento G, De Palma L (2021). The impact of covid-19 lockdowns on sustainable indexes. Sustainability (Switzerland).

[CR6] Cortez M, Leite P (2015). Performance of European Socially Responsible Funds during Market Crises: Evidence from France. Int Rev Financ Anal.

[CR7] Cortez M, Silva F, Areal N (2009) Socially Responsible Investing in the Global Market: The Performance of US and European Funds. Int J Finance Econ. 17 (3). 10.2139/ssrn.1342469

[CR8] Costa R, Vasconcelos G (2003) Long-range correlations and nonstationarity in the Brazilian stock market. Phys A 329(1-2):231–248

[CR9] Cunha FAFDS, de Oliveira EM, Orsato RJ, Klotzle MC, Cyrino Oliveira FL, Caiado RGG (2020). Can sustainable investments outperform traditional benchmarks? evidence from global stock markets. Bus Strateg Environ.

[CR10] Dahlhaus R (1989). Efficient parameter estimation for self-similar process. Ann Stat.

[CR11] de Dios-Alija T, del Río Caballero M, Gil-Alana L A, Martin-Valmayor M (2021) Stock market indices and sustainability: A comparison between them. J Sustain Finan Invest. 10.1080/20430795.2021.1896988.

[CR12] Durán-Santomil P, González L, Domingues R, Reboredo J (2019) e.11. 2972. 10.3390/su11102972.

[CR13] El Ghoul S, Karoui A (2016) Does Corporate Social Responsibility Affect Mutual Fund Performance and Flows?. J Bank Finan. 77. 10.1016/j.jbankfin.2016.10.009.

[CR14] Gil-Alana LA, Robinson PM (1997). Testing of unit root and other nonstationary hypotheses in macroeconomic time series. J Econ.

[CR15] Gladish B, Méndez-Rodríguez P, Mzali B, Lang P (2013) Mutual Funds Efficiency Measurement under Financial and Social Responsibility Criteria. J Multi-Criteria Decis Anal. 20. 10.1002/mcda.1494

[CR16] Gray R (2006). Does sustainability reporting improve corporate behaviour? Wrong question? Right time?. Account Bus Res.

[CR17] Greene MT, Fielitz BD (1977). Long-term dependence in common stock returns. J Financ Econ.

[CR18] Hurst HE (1951). Long-term storage capacity of reservoirs. Trans Am Soc Civ Eng.

[CR19] Jacobsen B (1995). Are stock returns long-term dependent? Some empirical evidence. J Int Financ Mark Inst Money.

[CR20] Jain M, Sharma GD, Srivastava M (2019) Can sustainable investment yield better financial returns: A comparative study of ESG indices and MSCI indices. Risks 7(1). 10.3390/risks7010015

[CR21] Junkus J, Berry T (2015). Socially responsible investing: A review of the critical issues. Manag Financ.

[CR22] KPMG (2020) KPMG Survey of Sustainability Reporting/. Retrieved from: https://home.kpmg/uk/en/home/insights/2020/12/kpmg-survey-of-sustainability-reporting-2020.html

[CR23] Leite P, Cortez M (2013). Style and Performance of International Socially Responsible Funds in Europe. Res Int Bus Financ.

[CR24] Lo AW (1991). Long-term memory in stock market prices. Econometrica.

[CR25] Lopez MV, Garcia A, Rodriguez L (2007) Sustainable development and corporate performance: a study based on the dow jones sustainability index. J Bus Ethics 75(3):285–300

[CR26] Managi S, Okimoto T, Matsuda A (2012). Do socially responsible investment indexes outperform conventional indexes?. Appl Financ Econ.

[CR27] Mandelbrot B (1972). Statistical methodology for nonperiodic cycles: From the covariance to R/S analysis. Ann Econ Soc Meas.

[CR28] Mynhardt RH, Plastun A, Makarenko I (2017). Market efficiency of traditional stock market indices and social responsible indices: the role of sustainability reporting. Invest Manag Financ Innov.

[CR29] Onali E, Goddard J (2011). Are European equity markets efficient? New evidence from fractal analysis. Int Rev Financ Anal.

[CR30] Peters EE (1991). Chaos and order in the capital markets: A new view of cycles, prices, and market volatility.

[CR31] Peters EE (1994). Fractal market analysis: Applying chaos theory to investment and economics.

[CR32] Rehman RU, Abidin MZU, Ali R, Nor SM, Naseem MA, Hasan M, Ahmad MI (2021). The integration of conventional equity indices with environmental, social, and governance indices: Evidence from emerging economies. Sustainability (Switzerland).

[CR33] Rehman RU, Zhang J, Uppal J, Cullinan C, Akram Naseem M (2016). Are environmental social governance equity indices a better choice for investors? an asian perspective. Business Ethics.

[CR34] Robinson PM (1994). Efficient tests of nonstationary hypotheses. J Am Stat Assoc.

[CR35] Robinson PM (1995). Gaussian semi-parametric estimation of long-range dependence. Ann Stat.

[CR36] Schröder M (2004). The Performance of Socially Responsible Investments: Investment Funds and Indices. Fin Markets Portfolio Mgmt.

[CR37] Sharma GD, Talan G, Bansal S, Jain M (2021). Is there a cost for sustainable investments: Evidence from dynamic conditional correlation. J Sustain Financ Invest.

[CR38] Statman M (2000). Socially Responsible Mutual Funds (corrected). Financ Anal J.

[CR39] Tripathi V, Kaur A (2020). Socially responsible investing: Performance evaluation of BRICS nations. J Adv Manag Res.

[CR40] Umar Z, Kenourgios D, Papathanasiou S (2020). The static and dynamic connectedness of environmental, social, and governance investments: International evidence. Econ Model.

[CR41] Van der Lugt CT, van de Wijs PP, Petrovics D (2020) Carrots & Sticks 2020 - Sustainability reporting policy: Global trends in disclosure as the ESG agenda goes mainstream. Global Reporting Initiative (GRI) and the University of Stellenbosch Business School (USB). Retriwed from: https://www.carrotsandsticks.net/media/zirbzabv/carrots-and-sticks-2020-interactive.pdf

[CR42] Varma A, Nofsinger J (2014). Socially Responsible Funds and Market Crises. J Bank Finance.

